# The adaptive potential of circular DNA accumulation in ageing cells

**DOI:** 10.1007/s00294-020-01069-9

**Published:** 2020-04-15

**Authors:** Ryan M. Hull, Jonathan Houseley

**Affiliations:** 1 SciLifeLab, Department of Microbiology, Tumor and Cell Biology, Karolinska Institutet, Solna, Sweden; 2 Epigenetics Programme, The Babraham Institute, Cambridge, UK

**Keywords:** Circular DNA, Extrachromosomal DNA, Double minutes, Extrachromosomal circular DNA, Ageing, Non-Mendelian inheritance

## Abstract

Carefully maintained and precisely inherited chromosomal DNA provides long-term genetic stability, but eukaryotic cells facing environmental challenges can benefit from the accumulation of less stable DNA species. Circular DNA molecules lacking centromeres segregate randomly or asymmetrically during cell division, following non-Mendelian inheritance patterns that result in high copy number instability and massive heterogeneity across populations. Such circular DNA species, variously known as extrachromosomal circular DNA (eccDNA), microDNA, double minutes or extrachromosomal DNA (ecDNA), are becoming recognised as a major source of the genetic variation exploited by cancer cells and pathogenic eukaryotes to acquire drug resistance. In budding yeast, circular DNA molecules derived from the ribosomal DNA (ERCs) have been long known to accumulate with age, but it is now clear that aged yeast also accumulate other high-copy protein-coding circular DNAs acquired through both random and environmentally-stimulated recombination processes. Here, we argue that accumulation of circular DNA provides a reservoir of heterogeneous genetic material that can allow rapid adaptation of aged cells to environmental insults, but avoids the negative fitness impacts on normal growth of unsolicited gene amplification in the young population.

From a human perspective, the concept of mutation appears purely negative, associated only with degeneration and cancer. However, all living organisms must maintain an appropriate mutation rate to survive: too high a rate leads to genome instability and degradation of vital functions, but conversely too little mutation strangles evolution by suppressing genetic diversity, preventing adaptation to new environmental challenges and condemning an organism to be out-competed. Evolutionary studies in *E. coli* and asexual yeasts reveal that strains with high mutation rates outperform those with low mutation rates, showing that mutation provides an adaptive advantage (Arjan et al. 1999; Desai et al. 2007; Wielgoss et al. 2013). However, increasing the mutation rate only improves adaptability up to a critical “error threshold”: above this, further increases reduce adaptability because fitness-enhancing mutations only account for a very small proportion of total mutations, and the accumulation of more frequent deleterious mutations becomes limiting for fitness (Eyre-Walker and Keightley 2007; Gerrish et al. 2013; Lynch et al. 1993; Sprouffske et al. 2018).

It is well understood that prokaryotes supplement chromosomal genetic material with circular DNA plasmids that deviate from normal rules of Mendelian inheritance and accelerate adaptation. In contrast, the evolutionary significance of circular DNA in eukaryotes has until recently been largely ignored, despite evidence for circular DNA in eukaryotic nuclei dating back over half a century (Cox et al. 1965; Hotta and Bassel 1965). Here, we examine the potential of circular DNA to accelerate adaptation in eukaryotes in general and ask whether the accumulation of particular circular DNA species during ageing in yeast enhances the potential of those species to confer adaptive phenotypes, providing a beneficial outcome of ageing in simple eukaryotes.

Circular DNA ranges in size from a few hundred base pairs (100–1000 bp microDNA) to several megabases (1–5 Mb ecDNA also known as double minutes) (Paulsen et al. 2018; Shibata et al. 2012; Turner et al. 2017). Circular DNA can derive from sites with little or no sequence homology, however, highly repetitive genomic regions, such as the ribosomal DNA (rDNA), telomeres, transposon remnants, and tandemly repeated genes are the largest producers of circular DNA at least in yeast (Moller et al. 2015; Sinclair and Guarente 1997). Double-strand break repair is the primary source of circular DNA from these repetitive regions although other formation mechanisms have been proposed and may well apply to circular DNA arising from non-repetitive regions (Hull et al. 2019; Park et al. 1999; Paulsen et al. 2018). The existence of circular DNA appears to be common amongst eukaryotes and has so far been reported in fungi, trypanosomes, worms, flies, frogs, mammals and plants (Beverley 1991; Cohen et al. 1999; Hotta and Bassel 1965; Koo et al. 2018; Shoura et al. 2017; Stanfield and Helinski 1976). In healthy humans, circular DNA from many genomic regions are detected in somatic tissue and also form as a side-product of V(D)J recombination (Moller et al. 2018; Serana et al. 2013), but conversely circular DNA is detected in approximately half of human cancers, is more prevalent in malignant than benign tumours, and is associated with poor prognosis (Fan et al. 2011; Koche et al. 2020; Turner et al. 2017).

The vast majority of circular DNA molecules lack centromeres and, in the absence of other mechanisms, will segregate randomly in mitosis. Therefore, the copy number of a circular DNA is not necessarily the same in each daughter cell after mitosis and can be substantially higher or lower than that of the parental cell. Because of this non-Mendelian character, simulations and experiments show that individual circular DNA species can accumulate very rapidly (Nathanson et al. 2014; Turner et al. 2017), and evolution via circular DNA can be highly advantageous compared to chromosomal change under selection for increased gene dosage (deCarvalho et al. 2018; Ubeda et al. 2014). Dramatic copy number amplification of driving oncogenes and drug resistance factors are frequent in cancer (Beroukhim et al. 2010; Corcoran et al. 2010; Frei et al. 1984; Katoh 2008; Little et al. 2011), and it is perhaps unsurprising that these amplifications commonly occur through accumulation of circular DNA (deCarvalho et al. 2018; Koche et al. 2020; Storlazzi et al. 2010; Turner et al. 2017; Vogt et al. 2004; Von Hoff et al. 1990; Wu et al. 2019). Similarly, as circular DNA is often absent from one daughter after division, a sub-population is readily selected for decreased gene dosage (Gresham et al. 2010; Haber and Schimke 1981; Nathanson et al. 2014).

In budding yeast, circular DNA molecules derived from the ribosomal DNA (ERCs) are highly focused in aged cells and are suggested to drive premature ageing and shortened lifespan (Borghouts et al. 2004; Sinclair et al. 1997). Various mutants with exaggerated ERC accumulation have a reduced lifespan, for example, cells lacking the DNA helicase Sgs1 accumulate more ERCs than wild-types and display many age-associated phenotypes in addition to a shortened lifespan (Kaeberlein et al. 2004; Sinclair et al. 1997). Conversely, loss of the replication fork blocking protein Fob1 decreases the formation of ERCs and extends the lifespan of mother cells by 30–40% (Defossez et al. 1999). In addition to ERCs generated from the rDNA locus, sensitive sequencing methods have identified almost 1800 circular DNA species in young populations, covering 23% of the budding yeast genome (Moller et al. 2015). The vast majority of these circular DNAs contain at least a fragment of a protein-coding gene, but unlike ERCs these species are largely sub-stoichiometric, such that any given cell will only contain a few circular DNA molecules that increase the copy number of genes encoded on those circular DNAs by 1 copy. Although such changes can be adaptively useful, in most situations and for most genes, this degree of gene amplification is likely to have minimal effect. Indeed, fitness assays under different challenging environments show that genes present on low copy plasmids confer fitness effects rarely and of much lesser magnitude than those on multi-copy plasmids (Payen et al. 2016). Therefore, although young yeast populations contain a wide diversity of circular DNA, it is only with substantial accumulation of any given circular DNA that major phenotypic effects are likely to manifest.

Circular DNAs including ERCs accumulate in aged yeast through a highly asymmetric mitotic segregation process that is very different from the random segregation observed in mammalian cells (Murray and Szostak 1983). During cell division in budding yeast, mother cells retain ERCs and other molecules considered harmful, thereby promoting daughter cell fitness (Mortimer and Johnston 1959; Sinclair and Guarente 1997). ERC retention in mother cells is mediated by attachment via SAGA and TREX2 to nuclear pore complexes that are largely retained by the mother cell (Denoth-Lippuner et al. 2014; Shcheprova et al. 2008). However, asymmetric inheritance alone is insufficient to mediate circular DNA accumulation as additional copies of the circular DNA must be generated. ERCs and many other circular DNA species carry active replication origins and are, therefore, replicated during the cell cycle. Once replicated, both copies are retained in the mother cell and so the copy number doubles, accounting for the massive abundance of ERCs in aged cells which increase genome size by over 50% (Cruz et al. 2018). Surprisingly, we discovered that replication is not required for all circles, as *CUP1* circular DNA does not replicate efficiently (despite carrying an annotated replication origin), and is instead generated from chromosomal DNA at such a high rate that simple asymmetric retention of the newly generated copies in the mother cell is sufficient for *CUP1* accumulation (Hull et al. 2019). However, many circular DNA species do not form at a high rate, nor contain active replication origins and, therefore, the copy number of these species in mother cells will remain static or decrease with age. A curious outcome of these differences in formation speed, replication capacity and asymmetric retention is that the diversity of circular DNA species observed in young cells is high, but the copy number of each individual circle and, therefore, the phenotypic impact is low. As cells age, only a subset of circular DNA can accumulate but that subset reaches higher copy numbers that will have a greater phenotypic effect, beneficial or not (Fig. 1, steps 1 and 2). In other words, the diversity of circular DNA decreases with age in yeast, which has been observed experimentally (Prada-Luengo et al. 2020), but the effects of the remaining circular DNA species will progressively increase.

**Fig. 1:**
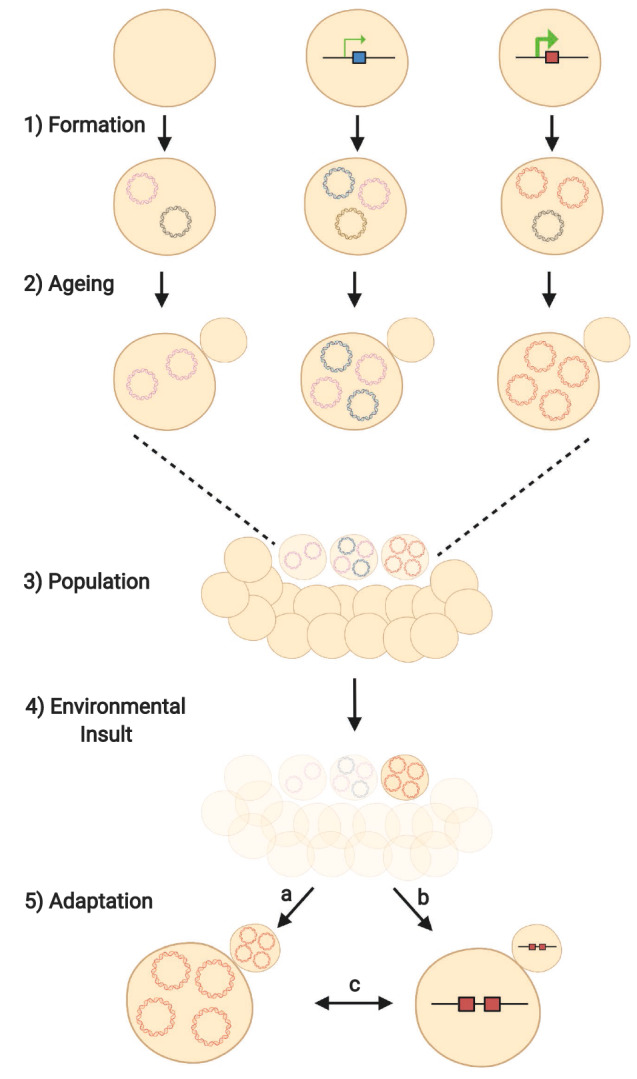
Rapid adaptation of aged cells to environmental insults. (1) Circular DNA molecules are formed from the linear chromosomes by both random (purple, black, and brown circles) and transcriptionally-stimulated (blue, and red circles) recombination and repair processes, producing a high diversity of low-copy circles in young cells. Highly-transcribed inducible genes (red box) produce more circles (red) than lowly-transcribed inducible genes (blue box and blue circles). (2) During ageing some circles (black and brown) are lost, whilst other circles (blue, purple and red) accumulate in mother cells by asymmetric segregation, creating a less-diverse but higher-copy circular DNA repertoire. (3) Circles are retained in the aged sub-population, which displays a fitness deficit, enabling the rest of the population of pre-dominantly young cells to grow unimpeded. (4) High-copy circles enhance adaptability in aged cells, increasing the likelihood of an aged cell having the necessary copy number mutation to survive an environmental insult. (5) The adaptive mutation is then propagated through the population by: **a** relaxing the asymmetric segregation of circular DNA and enabling donations to daughter cells; **b** re-integrating the beneficial circular DNA into the chromosome as a heritable duplication; **c** a combination of strategies **a** and **b**. Figure created with BioRender.com

The ageing yeast sub-population is, therefore, enriched for high-copy circular DNA containing protein-coding genes, and there are many examples of gene copy number accumulation providing adaptive advantages (Fig. 1, steps 3 and 4). For example, yeast can adapt to sulphate, nitrogen and glucose limitation, and to environmental toxins, through gene amplification (Beaupere and Labunskyy 2019; Brown et al. 1998; Fogel and Welch 1982; Gresham et al. 2008, 2010). Furthermore, a recent study in cancer cells revealed that circular DNA has an unusually open chromatin structure which would amplify the phenotypic effects of genes encoded on circular DNA relative to their chromosomal counterparts (Wu et al. 2019). Therefore, advantages exist in accumulating multiple copies of circular DNA encoding specific genes in certain environments (Fig. 1, step 4). However, there would be a fitness cost because unbalanced copy number amplification of most genes will impair gene regulatory networks and protein homeostasis to some extent. In other words, accumulation of multiple copies of random genes on circular DNA is more likely to be neutral at best or detrimental at worst, and it is not unreasonable to suggest that accumulation of genic circular DNA species may contribute to the age-related fitness decline currently attributed to ERCs.

Excitingly we have observed that the rate of copy number variation events including circular DNA formation is not completely random, which may improve this cost–benefit balance for aged cells (Hull et al. 2017, 2019). Formation of *CUP1* circular DNA, which encodes the copper resistance protein Cup1, is highly dependent on the transcription of the *CUP1* locus, which in turn is tightly regulated based on copper in the environment (Hull et al. 2019). Therefore, yeast ageing in the presence of copper transcribe *CUP1* and so produce many more copies of the *CUP1* circular DNA, which pre-adapts these cells to further increases in copper concentration. A link between transcriptional activity and circular DNA content has also been observed by the Regenberg lab, who found that per megabase of DNA, gene-rich chromosomes contribute more to the total level of circular DNA in healthy human tissues (Moller et al. 2018). Furthermore, *TTN* (titin), the most transcribed protein-coding gene in muscle tissue, is also the largest producer of circular DNA per gene (Moller et al. 2018). Formalising this idea, we suggest that genes which have evolved to be induced in response to particular environmental conditions are excellent candidates for adaptive amplification, and that simply connecting circular DNA formation to transcriptional induction is a clever means by which cells could gain the maximum chance of accumulating useful circular DNA, rather than unhelpful or negative species.

By itself, an adaptive phenotype in an individual aged cell is of little use if the causal circular DNA is selfishly retained in the mother cell, as would be the case if asymmetric segregation is maintained, and we must consider how circular DNA accumulation is translated into a heritable advantage. First, once circular DNA has accumulated, segregation can be relaxed under stress allowing circles with replication origins to propagate at high copy number in the population (Fig. 1, step 5a). This release of the asymmetric segregation system under heat stress has been observed and represents a general response to signalling from the cell wall integrity pathway (Baldi et al. 2017). Secondly, accumulation of high levels of circular DNA increases the chances of chromosome re-integration and, therefore, restoration of normal heritability for the amplified allele (Fig. 1, 5b). Such adaptive chromosomal re-integration events have been repeatedly observed, although it is unclear whether they happened in aged cells (Beverley et al. 1984; Brewer et al. 2015; Demeke et al. 2015; Durkin et al. 2012; Galeote et al. 2011; Koche et al. 2020; Lauer et al. 2018; Vogt et al. 2004).

The idea that a sub-population trades short-term growth for adaptive capacity is formalised in bet-hedging (concisely reviewed in (Levy et al. 2012)), and facets of ageing that fit with a bet-hedging model have been demonstrated experimentally including that aged yeast can be more stress resistant and more adaptable to nutrient transitions than young cells (Frenk et al. 2017; Levy et al. 2012). Cell-to-cell heterogeneity in the expression of metabolic enzymes and stress resistance factors also increases with age suggesting general phenotypic diversification of the ageing population (Radzinski and Reichmann 2019). These studies invoke transcriptional mechanisms linked to ageing to mediate switching to an adaptive state, whereas circular DNA excision represents a reversible genetic mechanism for phenotypic switching (Moller et al. 2013). The exciting feature of age-linked circular DNA accumulation in bet-hedging scenarios is that it forms a system that accrues diversity without impacting the genetic integrity of chromosomal DNA, but has the potential to be fixed rapidly in evolutionary timescales by chromosomal re-integration. Circular DNA, therefore, provides cells with the ability to explore the benefits of a high mutation rate, without the associated risk of generating deleterious chromosomal mutations, and with the possibility to make beneficial mutations permanent.
